# Five Mitochondrial Genomes of the Genus *Eysarcoris* Hahn, 1834 with Phylogenetic Implications for the Pentatominae (Hemiptera: Pentatomidae)

**DOI:** 10.3390/insects12070597

**Published:** 2021-06-30

**Authors:** Rongrong Li, Min Li, Jiang Yan, Ming Bai, Hufang Zhang

**Affiliations:** 1 Laboratory of Insect Evolution, Taiyuan Normal University, Jinzhong 030619, China; yanj@tynu.edu.cn (R.L.); limin12nk@163.com (M.L.); kks688@163.com (J.Y.); 2 Key Laboratory of Zoological Systematics and Evolution, Institute of Zoology, Chinese Academy of Sciences, Beijing 100101, China; 3 Xinzhou Teachers University, Xinzhou 034000, China

**Keywords:** mitogenome, *Eysarcoris*, Pentatominae, phylogenetic relationship

## Abstract

**Simple Summary:**

Pentatominae is the largest subfamily in the Pentatomidae, and most of its species are considered important agricultural pests. The phylogenetic relationships of tribes within Pentatominae remain controversial despite the fact that many studies have been performed using various molecular markers. In this study, five mitogenomes of the genus *Eysarcoris* were sequenced and analyzed, and the phylogenetic relationships of tribes within Pentatominae were reconstructed. The gene arrangement of the five mitochondrial genomes were found to be conserved and identical to other heteropteran mitogenomes. Differences in start codon usage and tandem repeats within control regions were found between *E. gibbosus* and the other four *Eysarcoris* species. In addition, the phylogenetic analyses showed that *E. gibbosus* is the first diverging clade within Eysarcorini. The results support the proposal to transfer *E. gibbosus* to the *Stagonomus*, another genus of Eysarcorini. Our results clarified the phylogenetic relationships among tribes of Pentatominae and laid a foundation for the further studies of Pentatominae.

**Abstract:**

Four complete mitogenomes of *Eysarcoris rosaceus*, *E. montivagus*, *E. gibbosus*, *E. annamita* and one near-complete mitochondrial genome of *E. ventralis* were sequenced and used to explore the phylogenetic relationships of tribes within the subfamily Pentatominae. The mitogenomes range from 15,422 to 16,043 base pairs (bp) in length and encode 37 genes, including 13 protein-coding genes (PCGs), two rRNA genes, 22 tRNA genes (21 in *E. ventralis*), and a control region. Similar to other heteropteran species, the AT contents of the sequenced species were higher than their GC contents. The most frequently used start/stop codon was ATN/TAA. GTG was only found in *atp6* and *atp8* of *E. gibbosus*. All transfer RNA genes (tRNAs) exhibit the typical cloverleaf secondary structure, except for the *trnS1* and *trnV*, which lacks the stem of the DHU arm. The length and copy number of repeat units were conserved within *Eysarcoris*, with the exception of *E. gibbosus*. Phylogenetic analyses based on mitogenomes using both maximum likelihood (ML) and Bayesian inference (BI) methods strongly supported the relationship among tribes within Pentatominae and confirmed that *Graphosoma* should be an intermediate lineage of Pentatominae. The relationship between *Eysarcoris* and *Carbula* was strongly supported and combined with our previous geometric morphometrics and chromosomal studies, suggest the *Eysarcoris* should belong to the tribe Eyasrcorini. This work will help to enhance our understanding of mitochondrial genomic evolution and phylogenetic relationships in Pentatominae.

## 1. Introduction

Pentatominae is the most diverse subfamily in Pentatomidae, and its members are found worldwide. It consists of at least 3484 species belong to 660 genera, in 43 tribes [1]. The lack of unique diagnostic characteristics hampers the identification of this subfamily, making it difficult to construct criteria for practical and reliable classification. As a result, the system used for classification of species in Pentatominae has varied across studies [1]. For example, *Eysarcoris* Hahn, 1834 (Hemiptera: Pentatomidae: Pentatominae) has been successively placed into four different tribes (Eusarcocoriaria, Pentatomini, Graphosomini and Eysarcorini) [1,2,3,4,5]. The scutellum of *Carbula* Stål 1864, a member of Eysarcorini, is not as large as in most eysarcorine genera, and Linnavuori [6] proposed a group including *Carbula* and six other genera. *Graphosoma* Laporte de Castelnau, 1833 was treated as a member of Graphosomini of Pentatominae in Yang’s nine-tribes classification system based on the scutellum shape [4], while another study placed it in Podopinae, another subfamily of Pentatomidae [1,5]. Most of the previous studies have focused on the high-level relationships within Heteroptera, while the phylogenetic relationships of tribes within Pentatominae remain controversial.

*Eysarcoris* is a genus of small, mottled brown shield bugs that are widely distributed in Europe, Asia, Africa, and Australia [7]. It is known as an important pest of upland rice, wheat, cotton, and soybean, among others, and injures crops by sucking fluid sap from the stem and grain, damaging plant health to such an extent that it can cause crop failure [8,9].

Members of *Eysarcoris* are usually small in size and tend to be robust in shape. The two small and smooth yellow or pale spots on the basal angle of the scutellum easily differentiate it from other genera of Pentatominae. However, members of *Eysarcoris* show inconspicuous inter- and intraspecific morphological differences [2,4]. Previously, geometric morphometric methods were employed to investigate the morphological variation within or among *Eysarcoris* species [10,11,12,13]. However, molecular studies using gene fragments (*cox1*, *16s rRNA*) have only been used to study the taxonomic status of *E. aeneus* (Scopoli, 1763) and *E. gibbosus* (Jakovlev, 1904) [14,15]. 

An insect mitochondrial genome is typically a double-stranded, circular DNA molecule ranging from 15 to 18 kb in size [16,17,18,19]. It is generally composed of 37 genes: 13 protein-coding genes (PCGs), two ribosomal RNA genes (rRNA), 22 transfer RNA genes (tRNA), and a control region (also known as the AT-rich region) of variable length that is thought to be essential in transcription and replication [16]. Due to its relatively small size, haploid nature, high rate of evolution, relatively conserved gene content, and organization, mitogenomes of insects have been widely used in species classification, population genetic structure, evolutionary biology, phylogenetic, and biogeographic studies [20,21,22,23,24,25,26]. So far, only 25 complete or near-complete mitogenomes of Pentatominae have been reported, which is a relatively small number considering its species richness. Furthermore, the phylogeny of the Pentatominae based on mitogenomes data is still limited.

In this study, we sequenced and compared the mitogenomes of *E. rosaceus* Distant, 1901, *E. montivagus* (Distant, 1902), *E. gibbosus*, *E. annamita* Breddin, 1909, and *E. ventralis* (Westwood, 1837) and reconstructed the phylogenetic relationships of tribes within Pentatominae using Bayesian inference (BI) and maximum likelihood (ML) methods. The mitogenomes from closely related species will improve the accuracy of the delimitation of mitochondrial gene and provide useful information on the molecular evolution and phylogenetic relationships in Pentatominae at a genomic level.

## 2. Materials and Methods

### 2.1. Taxon Sampling and Mitogenome Sequencing

The specimens used in this study were collected from fields in China either manually or using sweeping nets (Table S1). The samples were impregnated in 100% ethanol and stored at −20 °C. The genomic DNA for each of the species was extracted from thoracic muscles of a single specimen using a Genomic DNA Extraction Kit (BS88504, Sangon, Shanghai, China). The mitochondrial genome was then sequenced on an Illumina MiSeq platform using the whole-genome shotgun method (Personalbio, Shanghai, China). FastaQC was used to ensure the quality of data (https://www.bioinformatics.babraham.ac.uk/projects/fastqc/; accessed on 12 December 2020). Trimmomatic v0.36 was used to remove adapter sequences and low-quality bases (Q value < 20 and sequence length < 50 bp) [27]. A5-miseq v20150522 and SPAdes v3.9.0 were used for mitochondrial genome assembly [28,29].

### 2.2. Genome Annotation and Sequence Analysis

The Geneious v9.1.4 software (Biomatters Ltd., San Diego, CA, USA) was used to annotate the five newly sequenced mitogenomes [30]. The 13 PCG boundaries were identified by ORF finder on the NCBI website applying the invertebrate mitochondrial code (http://www.ncbi.nlm.nih.gov/orffinder/; accessed on 25 December 2020). tRNA genes were identified using the MITOS web server [31]. rRNAs were aligned with the previously sequenced mitochondrial sequences to confirm the accuracy of gene boundaries. The exact location of the control region was determined by confirming the boundary of neighboring genes. 

Nucleotide composition and codon usage were analyzed using MEGA-X [32]. The strand asymmetry was calculated as follows: AT skew = (A − T)/(A + T); GC skew = (G − C)/(G + C). The number of non-synonymous substitutions per nonsynonymous site (Ka) and synonymous substitutions per synonymous site (Ks) for each PCG was calculated using DnaSP 6, with exclusion of stop codons and codons with alignment gaps [33]. Tandem repeats in control regions were predicted using the Tandem Repeats Finder web server [34].

### 2.3. Phylogenetic Analyses

The phylogenetic analyses were conducted using the five newly sequenced mitochondrial genomes as well as those of 25 Pentatominae taxa, *Graphosoma rubrolineatum* (Westwood, 1837), and two Asopinae species (used as the outgroup) (Table S2). Plugins in Phylosuite v1.2.2 were used to prepare the datasets (13 PCGs and 2 rRNAs) for the phylogenetic analyses, which were conducted using BI and ML methods [35]. The alignment of PCGs/rRNAs was conducted using MAFFT according to a codon-based/normal alignment model, and gaps and ambiguous sites were then removed by Gblocks [36,37]. All alignments were then concatenated into a single data matrix using the concatenate sequence function in Phylosuite v1.2.2. The best-fit partitioning strategy and models of the concatenated sequences for BI and ML tree were selected by ModelFinder installed in Phylosuite v1.2.2 and model selection in the IQ-TREE web server [38]. The results were used to reconstruct the phylogenetic tree by Mrbayes installed in Phylosuite v1.2.2 and the IQ-TREE web server using Bayesian and ML methods, respectively [39,40]. For the Bayesian method, GTR + F + I + G4 was chosen as the best-fit model. Four independent Markov chains (three heated and one cold) were run for 10,000,000 generations and trees were sampled every 1000 generations. The first 25% of samples were discarded as a burn-in when the average standard deviation of split frequencies > 0.01. For the ML method, GTR + R5 + F was the best-fit model, and the analysis was assessed under ultrafast replications (1000).

## 3. Results

### 3.1. Mitochondrial Genomic Structure

**Figure 1:**
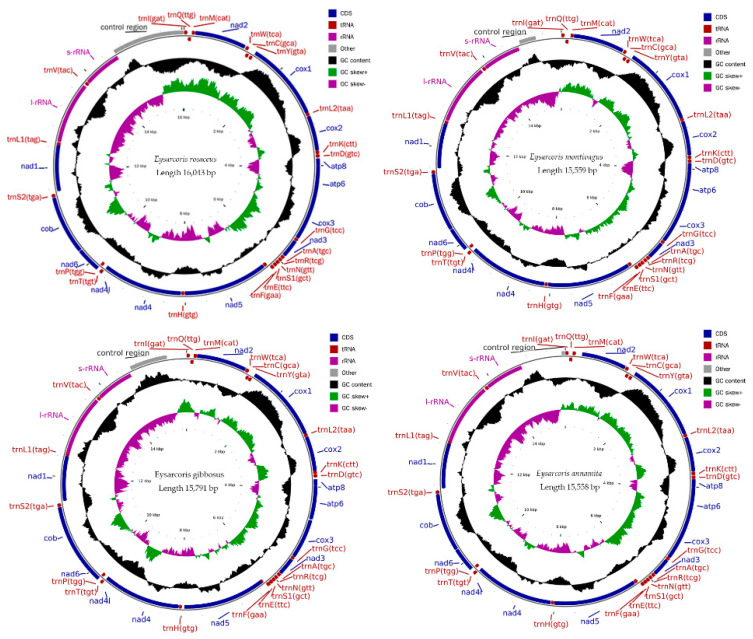
Gene arrangements of the four complete mitochondrial genomes.

The mitochondrial genomes of *E. rosaceus, E. montivagus, E. gibbosus*, and *E. annamita* are circular double-stranded molecules ranging from 15,558 to 16,043 bp in length (Figure 1). The mitogenome of *E. ventralis* lacks the *trnV* gene and is 15,422 bp long (Figure S1). The complete mitogenome sequences encode a complete set of 37 genes, which is also the case for most other heteropteran mitogenomes, and includes 13 PCG, 2 rRNAs, 22 tRNAs, and a noncoding control region (putative control region) (Table S3). Gene arrangement of the mitochondrial genomes is conserved, with 23 genes locate on the J-strand and 14 genes (13 in *E. ventralis*) on the N-strand (Table S3). 

A conserved 2 bp gene spacer was observed between *nad4L* and *trnT*. The gene spacer was 1 bp long between *trnV* and *12s rRNA* in the four complete mitochondrial genomes. The total length of intergenic spacers ranged from 88 to 167 bp, and the longest single spacer was 31 bp long, observed in *E. montivagus* between *trnM* and *trnQ*. There were also conserved gene overlaps in the five mitogenomes, including *trnW*/*trnC* (8 bp), *cox1*/*trnL2* (5 bp), *atp8*/*atp6* (7 bp) and *trnN*/*trnS* (1 bp) (Table S3). 

For the examined species, the nucleotide composition of the whole mitogenome, PCGs, tRNAs, and rRNAs all showed high AT nucleotide content and low variability (Table S4). The AT-skew values of PCGs−, tRNAs−, and rRNAs were negative, while that of the whole mitogenome, PCGs+, and tRNAs+ were positive (Table S4). For GC skew, negative values were only found in the whole mitogenome and PCGs+ (Table S4).

### 3.2. Protein-Coding Genes

For all of the five studied species, nine PCGs (*nad2*, *cox1*, *cox2*, *atp8*, *atp6*, *cox3*, *nad3*, *nad6*, and *cytb*) were found to be coded on the majority strand (J-strand) and four PCGs (*nad5*, *nad4*, *nad4L*, and *nad1*), on the minority strand (N-strand). The longest PCG is *nad5* (1705–1710 bp), while the shortest is *atp8* (150–162 bp). The AT-skew values of *cytb*, *nad1*, *nad4*, *nad4L* and *nad5* are negative, while GC-skew of *nad1*, *nad4*, *nad4L* and *nad5* are all positive for the five mitogenomes. Two PCGs—*nad4* and *nad4L*—did not vary in length among the five species (Table S3). Most of the PCGs use an ATN (ATT/ATA/ATG/ATC) initiation codon. TTG was the second most used initiation codon, and was found in *cox1*, *atp8* (except in *E. gibbosus*), and *nad6*. In particular, only *atp8* and *atp6* in *E. gibbosus* use GTG as the initiation codon. The coding region of most PCGs ends with the complete termination codon TAA, except for *cox2* (in *E. rosaceus*, *E. montivagus*, *E. annamita*, and *E. ventralis*), *atp6* (in *E. annamita*), *cox3* (in *E. ventralis*), *nad3* (in *E. montivagus*), and *nad5* (in *E. gibbosus*), which ended with the incomplete stop codon T. 

**Figure 2:**
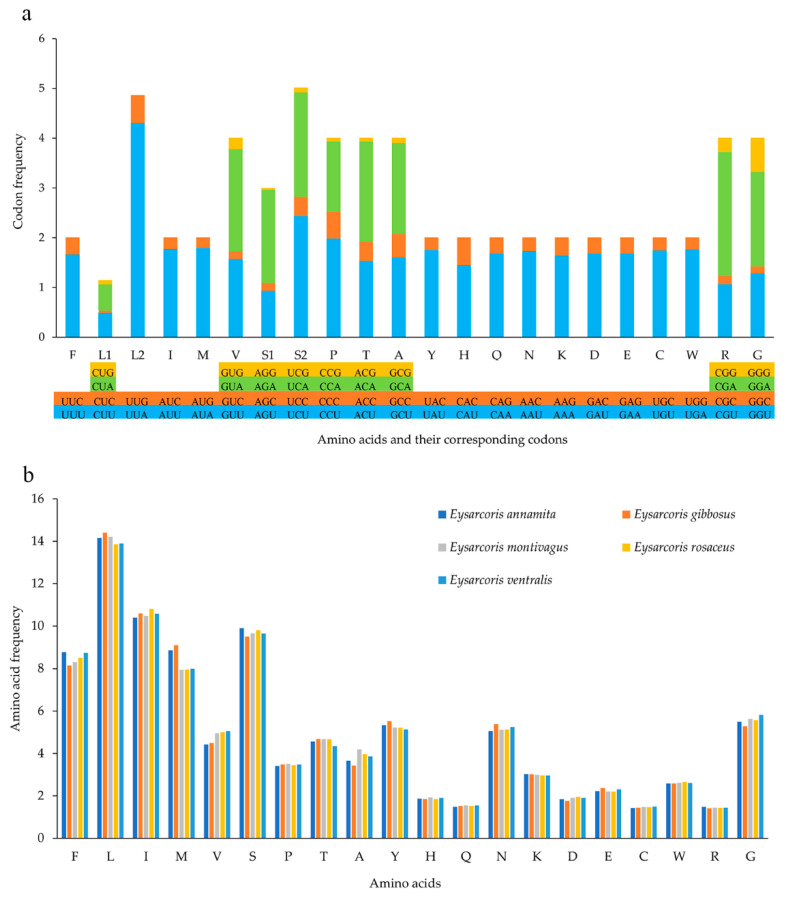
The frequencies of utilized codons by amino acid (**a**) and the frequency of amino acid occurrence (**b**) in the five newly sequenced mitochondrial genomes. The stop codon is not given.

The 13 PCGs of the five mitogenomes were found to consist of 3668 codons on average and showed very strong biases in amino acid composition and codon usage. Leu, Ile, Ser, Phe, and Met were the most abundant amino acids, while TTA (Leu), ATT (Ile), UCU (Ser) TTT (Phe), and ATA(Met) are the most frequently utilized codons (Figure 2). The most frequently utilized codons were composed of A and T, except UCU (Ser). As shown in Figure 2, there was a preference for use of A or T in the third position of codons, rather than G or C.

**Figure 3:**
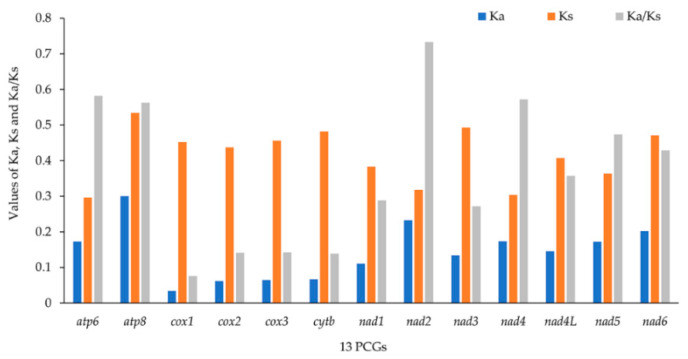
The Ka (the number of non-synonymous substitutions per nonsynonymous site), Ks (the number of synonymous substitutions per synonymous site) and Ka/Ks values of protein-coding genes within Pentatominae.

The values of Ka, Ks and Ka/Ks were calculated for each PCG to investigate the evolutionary patterns among mitochondrial PCGs in Pentatominae (Figure 3). The Ka/Ks ratio for all 13 PCGs were below 0.73, indicating evolution under purifying selection. The Ka/Ks ratio of *nad2* was the highest, while that of *cox1* was the lowest. We also observed lower Ka/Ks ratios in the genes that are usually used as a barcode, such as *cox2*, *cox3*, and *cytb*.

### 3.3. Transfer and Ribosomal RNAs

**Figure 4:**
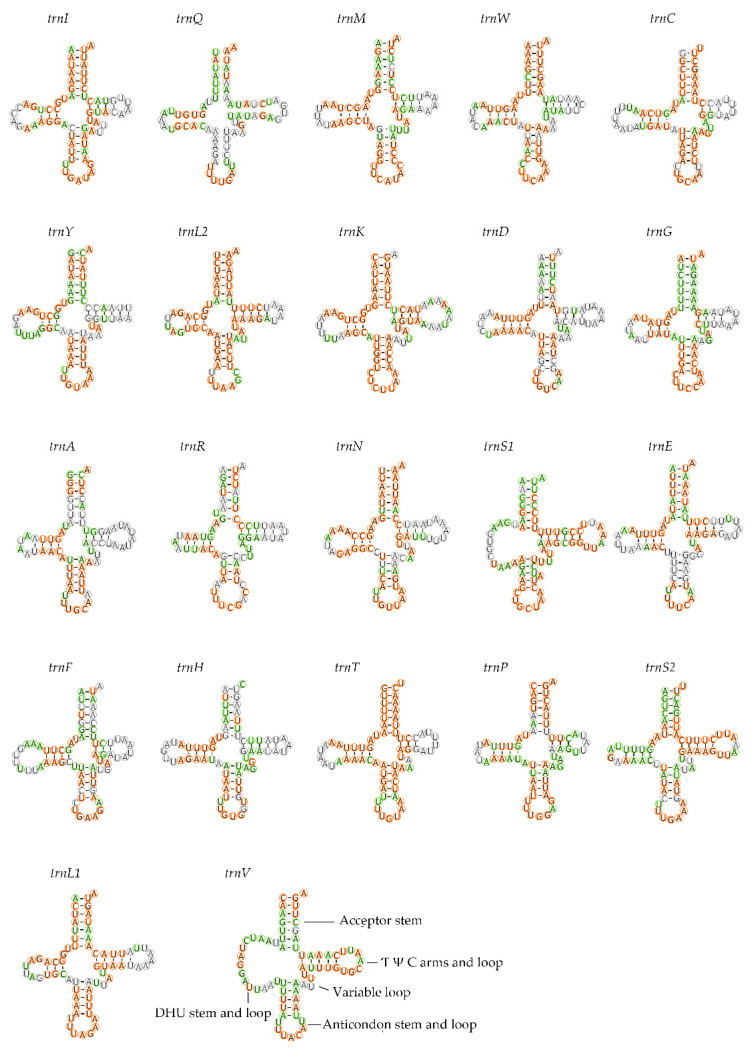
Potential secondary structure of tRNA in *Eysarcoris rosaceus*. The sites conserved within *Eysarcoris* are labeled in green, and those of Pentatominae are marked in orange.

All 22 of the typical tRNA genes for the five mitogenome ranged from 62 to 74 bp in length. Fourteen tRNA genes (*trnI*, *trnM*, *trnW*, *trnL2*, *trnK*, *trnD*, *trnG*, *trnA*, *trnR*, *trnN*, *trnS1*, *trnE*, *trnT*, *trnS2*) are coded on the majority strand and eight (*trnQ*, *trnC*, *trnY*, *trnF*, *trnH*, *trnP*, *trnL1*, *trnV*) on the minority strand. The arrangement of tRNA genes for the five mitogenomes was similar. Among all tRNAs, twenty had the typical cloverleaf structure, and two—*trnS1* and *trnV*—lacked the dihydrouridine (DHU) arm and form a loop. All tRNAs in the five mitogenomes use the standard anticodon. The sequences and structures of anticodon arms and aminoacyl acceptor stems were well conserved within Pentatominae, whereas most of the variations (nucleotide substitutions and indels) were found in the DHU loops, pseudouridine (TΨC) arms, and variable loops (Figure 4).

**Figure 5:**
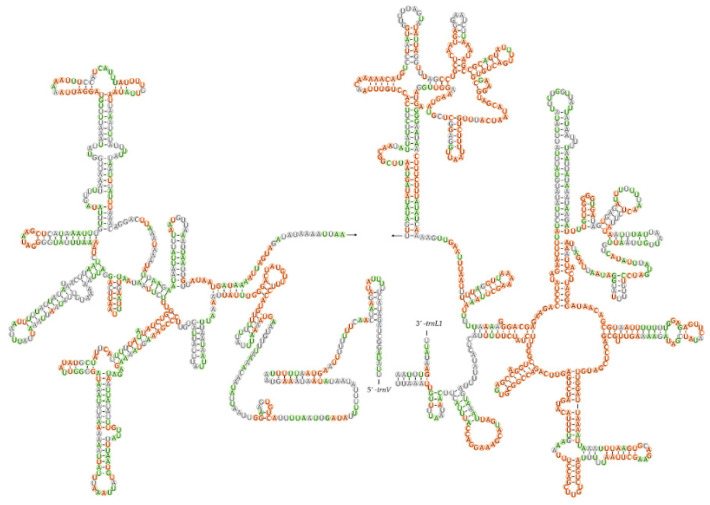
Potential secondary structure of 16S rRNA in *Eysarcoris rosaceus*. The sites conserved within *Eysarcoris* are labeled in green, and those of Pentatominae are marked in orange.

**Figure 6:**
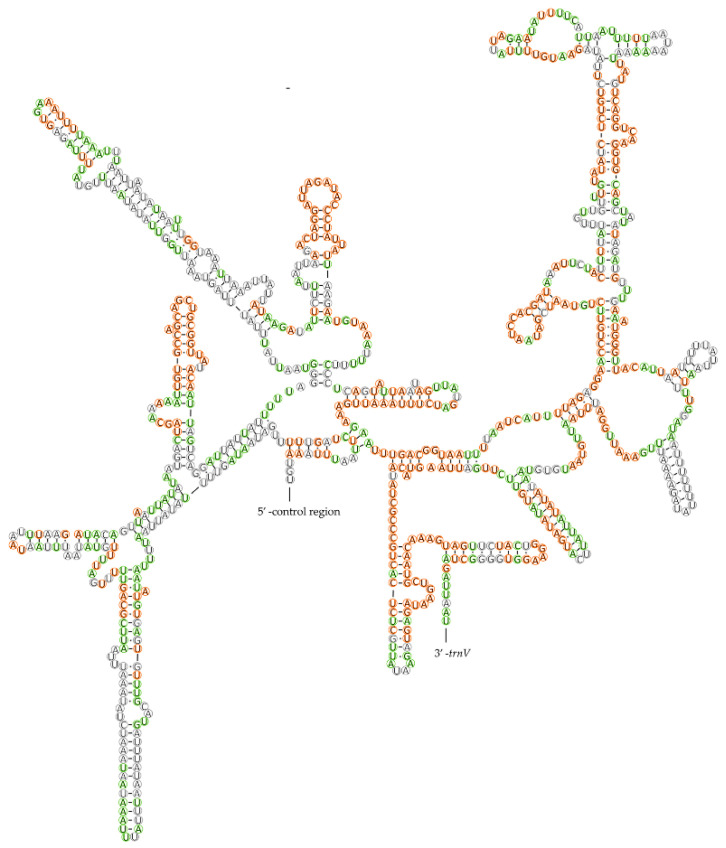
Potential secondary structure of 12S rRNA in *Eysarcoris rosaceus*. The sites conserved within *Eysarcoris* are labeled in green, and those of Pentatominae are marked in orange.

Two rRNA genes—*16s rRNA* and *12s rRNA*—were found on the minority strand in the five mitogenomes. The *16s rRNA* gene, ranging from 1246–1277 bp in size, is located at a conserved position between *trnL1* and *trnV*. The *12s rRNA* (785–795 bp) was found between *trnV* and the control region. The secondary structures of *16s rRNA* and *12s rRNA* were also predicted and are shown in Figure 5 and Figure 6, respectively. In the six domains of *16s rRNA*, domain IV and the 3′-end of domain V were more conserved within Pentatominae than domains I, II, and VI (Figure 5). The secondary structure of *12s rRNA* comprised three structural domains, and the stem region of domain III was structurally more conserved than domains I and II (Figure 6).

### 3.4. Control Region

**Figure 7:**
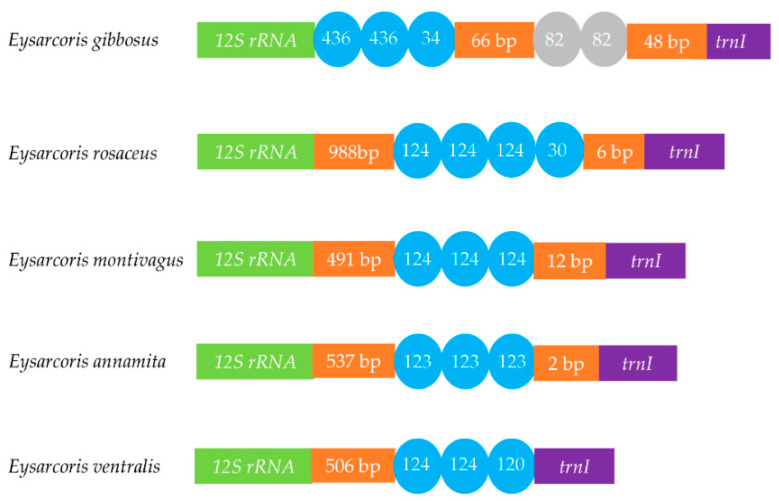
Organization of the control region in the five mitochondrial genomes. The tandem repeats are shown by the blue or gray oval with repeat length inside. Non-repeat regions are shown by orange box with sequence length inside.

The control regions are located between *12s rRNA* and *trnI*, and vary in length from 870 to 1396 bp. A comparison of tandem repeats in the control region of the five species is shown in Figure 7. The length and copies of repeat units differed among the five species. Only one type of tandem repeat was observed in *E. rosaceus*, *E. montivagus*, *E. annamita*, and *E. ventralis*. The tandem repeats in the control region of the four mitochondrial genomes are approximately 124 bp long. Two types of tandem repeats were found in *E. gibbosus*, with a 66 bp non-repeat region between them.

### 3.5. Phylogenetic Relationships

**Figure 8:**
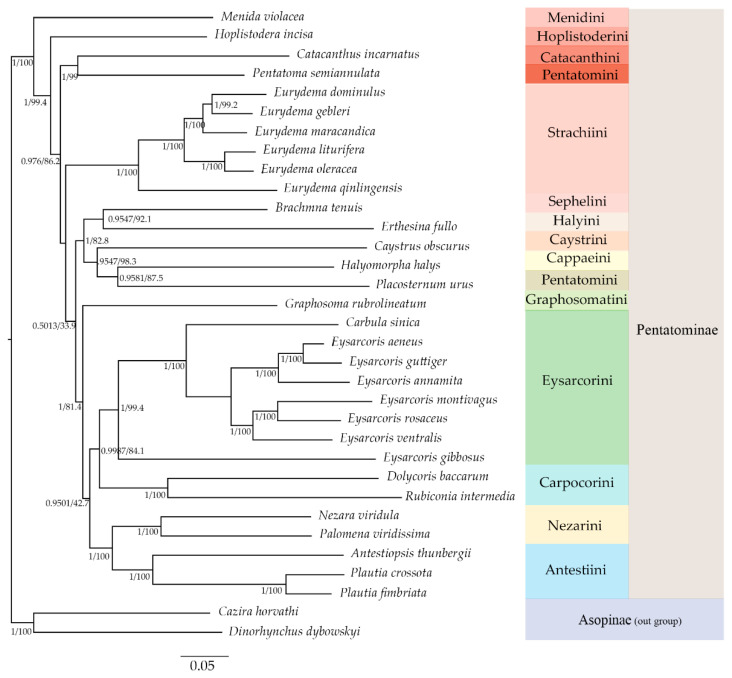
The phylogenetic relationships of tribes within Pentatominae reconstructed from DNA sequences of 13 protein coding and 2 rRNA mitochondrial genes using BI and ML methods.

Phylogenetic analyses were performed using BI and ML methods. The phylogenetic relationships among tribes within Pentatominae were reconstructed based on the sequences of the 13 PCGs and 2 rRNA genes. The results show that applying the two methods using the same dataset resulted in highly congruent tree topologies (Figure 8). The phylogenetic relationships of the Pentatominae were reconstructed, and the topology was as follows: (Menidini + (Hoplistoderini + ((Catacanthini + *Pentatoma semiannulata* (Motschulsky, 1859)) + (Strachiini + (((Sephelini + Halyini) + (Caystrini + (Cappaeini + *Placosternum urus* Stål, 1876))) + (Graphosomatini + ((Eysarcorini + Carpocorini) + (Antestiini + Nezarini)))))))). In Pentatominae, species of Carpocorini and Eysarcorini constituted one clade with high support values. *Graphosoma rubrolineatum* was sister to the clade ((Eysarcorini + Carpocorini) + (Antestiini + Nezarini)). The species of Pentatomini were divided into two clades, where *P. semiannulata* was a sister of Catacanthini, and *P. urus* was a sister of Cappaeini. For both of the BI and ML methods, *E. gibbosus* was found to be the first diverging clade within Eysarcorini, whereas *Carbula sinica* Hsiao & Cheng, 1977 was clustered to other species of *Eysarcoris*, which were divided into two clades: *E. annamita*, *E. aeneus*, and *E. guttiger* formed one clade and *E. ventralis*, *E. rosaceus*, and *E. montivagus* formed the other.

## 4. Discussion

In this paper, we describe the mitochondrial genomes of *E. rosaceus*, *E. montivagus*, *E. gibbosus*, *E. annamita*, and *E. ventralis*. Similar to that of other hemipteran mitogenomes, the gene arrangements of the five mitochondrial genomes are conserved [41,42,43,44,45,46,47]. The size of the complete mitogenome sequences varied widely among the examined species, ranging from 15,558 bp in *E. gibbosus* to 16,043 bp in *E. rosaceus* (Table S3). This variation in length has also been observed in other hemipteran mitogenomes and is primarily due to the significant size variation of the control region [41,42]. The comparison of tandem repeats in the control region seems to be conserved within *Eysarcoris*, with the exception of *E. gibbosus*, which had two types of tandem repeat. Previous studies have reported varying sizes and differentiated tandem repetitions in other Pentatomidae species [41,44]. Compared to the varied size of complete mitogenomes, relatively little variation was observed in the length of PCGs, tRNAs, and rRNAs. This is consistent with previous Pentatomidae mitochondrial genomic studies [41,43]. Similarly, the AT content of Pentatomidae species was significantly higher the GC content [26,41,42,43,44,45,46,47]. 

The most frequently occurring start codon of the five mitogenomes was found to be ATN, which is similar to most Pentatomidae mitogenomes [41,42,43,44]. Another frequently used start codon, TTG, usually appeared in the *cox1*, *atp8*, *nad1*, and *nad6* genes of Pentatomidae species [43]. We found that the use of TTG was conserved within the genus *Eysarcoris*, except for *E. gibbosus* (Table S1). Another start codon, GTG, is rarely used as a start codon in Pentatominae, and has only been reported in *E. gibbosus* in this study and in *P. semiannulata* in a previous study [44]. Regarding the stop codon, most PCGs ended with TAA or TAG. The use of the truncated stop codon T in *cox2* is conserved in Pentatominae (except *E. gibbosus*), while in *atp6*, *cox1*, *cox3*, *nad3*, *nad4*, *nad5*, and *nad6*, the choice of stop codon seems more diverse [43]. 

In the five mitogenomes we sequenced, the majority of tRNAs were found to have a canonical cloverleaf secondary structure. However, *trnS1* and *trnV* lack the stem of the DHU arm, and the loss of the DHU arm in trnS1 (AGN) has been considered a typical feature of insect mitogenomes [48]. We found that the anticodon arm and the amino acid acceptor stem are highly conserved in Pentatominae. 

In this study, the phylogenetic analysis based on PCGRNA matrix strongly supported the relationship between *Carbula* and *Eysarcoris*, and they formed an independent clade with high support values. This result was consistent with previous geometric morphometrics and chromosomal studies [12,49]. We also found that *E. gibbosus* was the first diverging clade within Eysarcorini. This is in accordance with a previous study based on *16s rRNA* and *cox1* genes [15]. Combined with the control region structure and start codon usage, we support the proposal to transfer *E. gibbosus* to the genus *Stagonomus* [15]. The phylogenetic analysis indicates that *Eysarcoris* and *Carbula* belong to Eysarcorini. 

There is little to separate Eysarcorini from Carpocorini, except for the genera with an enlarged scutellum [1]. Some genera of Carpocorini, such as *Rubiconia* Dohrn, 1860, were once transferred to Eysarcorini [50]. Our study shows that *R. intermedia* is a sister of *Dolycoris baccarum* (Linnaeus, 1758), another member of Carpocorini, and this is in accordance with the latest morphological classification and mitochondrial analysis [1,26]. The taxonomic status of *Graphosoma* was also ambiguous [1,4,5]. Our phylogenetic analysis revealed that *G. rubrolineatum* represents an intermediate lineage of Pentatominae, which corresponds with the findings in previous studies [26,44] and may indicate that *Graphosoma* is a genus within Pentatominae. Pentatomini is one of the more poorly defined tribes within Pentatominae. Genera with an armed abdominal venter and showing no characteristics of other tribes have been placed in Pentatomini, and *Placosternum* Amyot & Serville, 1843 is one of these genera [1]. The sternal structure is similar to that found in the Pentatomini, but the ostiolar rugae are much shorter or auriculate [1]. The difference in morphology and the phylogenetic results in this study may indicate that *Placosternum* should not belong to Pentatomini. 

Another two stable clades were observed in the phylogenetic analysis with high support values. Clade I comprised Nezarini and Antestini. The possible relationship between Nezarini and Antestiini was discussed by Gross and Linnavuori based on their morphological resemblance, and this was confirmed by our mitochondrial phylogenetic analysis and that of a previous study [6,51]. Sephelini, Halyini, Caystrini, Cappaeini, and *P. urus* formed clade II. *Eurydema* Laporte, 1833 was supported to be sister to Pentatomini in previous study [43]. However, Pentatomini was clustered to Catacanthini with height support value in this study. The support values between the genus *Eurydema* and its sister clades were lower than others, possibly because of the limited number of mitogenomes used in this study, and further studies should be carried out to resolve this. 

In previous studies, high-level relationships within the Heteroptera have received much attention, whereas there has been comparatively limited research conducted on the phylogenetic relationships of tribes within subfamilies. In the present study, five mitogenomes from the Pentatominae were sequenced and added to the pool of existing data. Our findings reveal the relationships among tribes within Pentatominae, and more mitogenomes should be sequenced to comprehensively understand the mitogenomic evolution and phylogenetic relationships of Pentatominae.

## Data Availability

The data supporting the findings of this study are openly available in National Center for Biotechnology Information (https://www.ncbi.nlm.nih.gov accessed on 27 June 2021), accession numbers were MT165687, MW846867, MW846868, MW852483, and MT165688.

## Supplementary Materials

The following are available online at https://www.mdpi.com/article/10.3390/insects12070597/s1. Figure S1: Mitochondrial genome map of *E. ventralis*. Table S1: Information of the specimens used in this study. Table S2: List of sequences used to reconstruct the phylogenetic relationships within Pentatominae. Table S3: Annotation and organization of the five *Eysarcoris* mitochondrial genomes. Table S4: AT skew and GC skew of the all the mitogenomes sequenced in the present study.
