# Supplementary figures and images for: Five Mitochondrial Genomes of the Genus Eysarcoris Hahn, 1834 with Phylogenetic Implications for the Pentatominae (Hemiptera: Pentatomidae)
Source: Insects. 2021 Jun 30;12(7):597. doi: 10.3390/insects12070597 (PMC8306050; doi:10.3390/insects12070597)

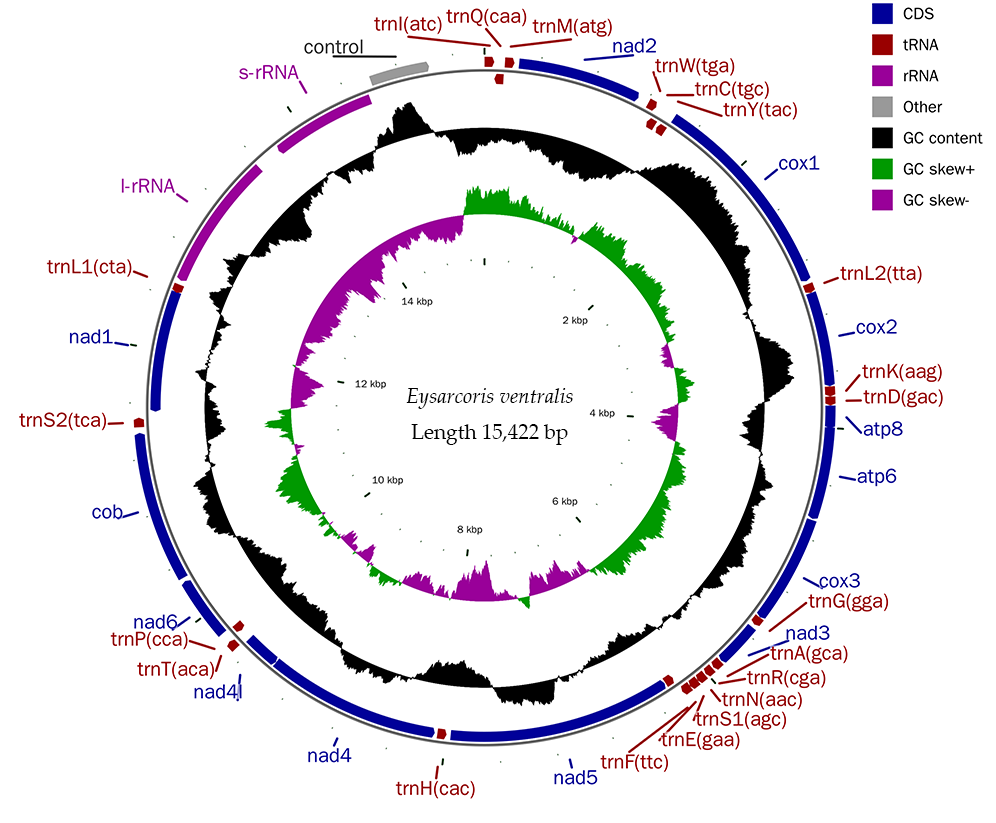

Supplement: Supplementary file 1 [file insects-12-00597-s001.zip › FigureS1.tif]
